# Expression and functional analysis of the plant-specific histone deacetylase *HDT701* in rice

**DOI:** 10.3389/fpls.2014.00764

**Published:** 2015-01-20

**Authors:** Jinhui Zhao, Jianxia Zhang, Wei Zhang, Kunlin Wu, Feng Zheng, Lining Tian, Xuncheng Liu, Jun Duan

**Affiliations:** ^1^Key Laboratory of South China Agricultural Plant Molecular Analysis and Genetic Improvement, South China Botanical Garden, Chinese Academy of SciencesGuangzhou, China; ^2^University of Chinese Academy of Sciences, Chinese Academy of SciencesBeijing, China; ^3^Southern Crop Protection and Food Research Centre, Agriculture and Agri-Food CanadaLondon, ON, Canada

**Keywords:** HDT701, histone deacetylase, seed germination, abiotic stress, gene expression

## Abstract

Reversible histone acetylation and deacetylation at the N-terminus of histone tails play a crucial role in regulating eukaryotic gene activity. Acetylation of core histones is associated with gene activation, whereas deacetylation of histone is often correlated with gene repression. The level of histone acetylation is antagonistically catalyzed by histone acetyltransferases citation(HATs) and histone deacetylases (HDACs). In this work, we examined the subcellular localization, expression pattern and function of *HDT701*, a member of the plant-specific HD2-type histone deacetylase in rice. HDT701 is localized at the subcellular level in the nucleus. Histochemical GUS-staining analysis revealed that *HDT701* is constitutively expressed throughout the life cycle of rice. Overexpression of *HDT701* in rice decreases ABA, salt and osmotic stress resistance during seed germination. Delayed seed germination of *HDT701* overexpression lines is associated with decreased histone H4 acetylation and down-regulated expression of GA biosynthetic genes. Moreover, overexpression of *HDT701* in rice enhances salt and osmotic stress resistance during the seedling stage. Taken together, our findings suggested that *HDT701* may play an important role in regulating seed germination in response to abiotic stresses in rice.

## Introduction

In eukaryotic cells, gene activity is controlled not only by the DNA sequence but also by epigenetic events. Switch of the chromatin structure between the condensed status and the relaxed status plays an important role in the regulation of gene expression (Goldberg et al., 2007). Post-translational histone changes lead to an alteration of DNA activity by histone acetylation, methylation, phosphorylation, ubiquitination, and sumoylation (Berger, 2007). The reversible acetylation or deacetylation of specific lysine residues at the N-terminal of histone tails are catalyzed by histone acetyltransferases citation(HATs) and histone deacetylases citation(HDACs) (Pandey et al., 2002). In general, hyper-acetylation of the histones is associated with gene activation whereas the hypoacetylation of histones is correlated with transcriptional repression. Plant HDACs can be grouped into three major families, the RPD3/HDA1 superfamily, the SIR2 family, and the HD2 family (Pandey et al., 2002).

HD2 proteins are plant-specific HDACs. HD2 proteins were first identified in maize as an acidic nucleolar phosphoprotein in a high molecular weight complex (Brosch et al., 1996; Lusser et al., 1997). Four HD2 proteins, HD2A (also known as HDT1), HD2B (HDT2) (Wu et al., 2000), HD2C citation(HDT3) (Zhou et al., 2004), and HD2D (HDT4) have been identified in *Arabidopsis* (Dangl et al., 2001; Pandey et al., 2002). The functions of the HD2 family members have been well-studied in recent years. It was demonstrated that HD2A, HD2B, and HD2C are able to mediate transcriptional repression (Wu et al., 2000, 2003). Knockdown of *HD2A* in *Arabidopsis* results in seed abortion (Wu et al., 2003) and causes reduced silencing of *Arabidopsis* rDNA (Lawrence et al., 2004). *HD2C* is involved in abscisic acid (ABA) and salt and drought stress responses (Sridha and Wu, 2006; Luo et al., 2012a). Knockdown of *HD2A* and *HD2B* in the *asymmetric leaf1* citation(*as1*) or *as2* mutant background causes the formation of abaxialized and filamentous leaves, which suggests the involvement of HD2A and HD2B during leaf development in *Arabidopsis* (Ueno et al., 2007).

Phylogenic analysis of the rice genome suggests there are at least two members of HD2 genes, *HDT701* and *HDT702*, in rice (Fu et al., 2007). Knockdown of *HDT702* rice plants displayed severely narrowed leaves, suggesting that *HDT702* might be involved in cell division or growth (Hu et al., 2009). Furthermore, overexpression of *HDT701* in transgenic rice leads to decreased levels of histone H4 acetylation and enhanced susceptibility to rice pathogens *Magnaporthe oryzae* and *Xanthomonas oryzae* pv *oryzae*, while silencing of *HDT701* in transgenic rice causes the levels of histone H4 acetylation and transcription pattern recognition of receptor and defense-related genes to become elevated, suggesting that *HDT701* is a histone H4 deacetylase and acts as a negative regulator in plant innate immunity in rice (Ding et al., 2012).

In this study, we investigated the subcellular localization, expression pattern and function of *HDT701* in rice. Our results suggested that *HDT701* may play essential roles in the regulation of seed germination in response to abiotic stresses.

## Results

### Cis-acting element analysis of *HDT701* and *HDT702* promoter sequences

We analyzed the *cis*-acting elements of the promoter regions of *HDT701* and *HDT702*. A 2000 bp DNA sequence before the start codon was used for the prediction using the PlantCARE database (Lescot et al., 2002). As shown in Supplemental Table 1, a large subset of stress-responsive *cis*-acting DNA regulatory elements was identified in *HDT701* and *HDT702* promoters. The following *cis*-acting elements were found in the promoters of *HDT701* and *HDT702*, respectively: 18 and 14 dehydration-responsive, 8 and 22 early responsive to dehydration, 8 and 11 pathogenesis-related, 4 and 4 disease-resistant responsive, and 2 and 8 ABA-responsive. This suggests that *HDT701* and *HDT702* may play an important role in the response to abiotic and biotic stresses. Furthermore, 20 and 9 cytokinin-related (CK), 2 and 2 salicylic acid (SA)-induced, and 1 and 3 jasmonate acid (JA)-responsive elements were also identified in the promoters of *HDT701* and *HDT702*, respectively. This indicates that *HDT701* and *HDT702* may be involved in multiple hormone signaling pathways.

### Expression patterns of *HDT701* and *HDT702* under abiotic stresses

**Figure 1:**
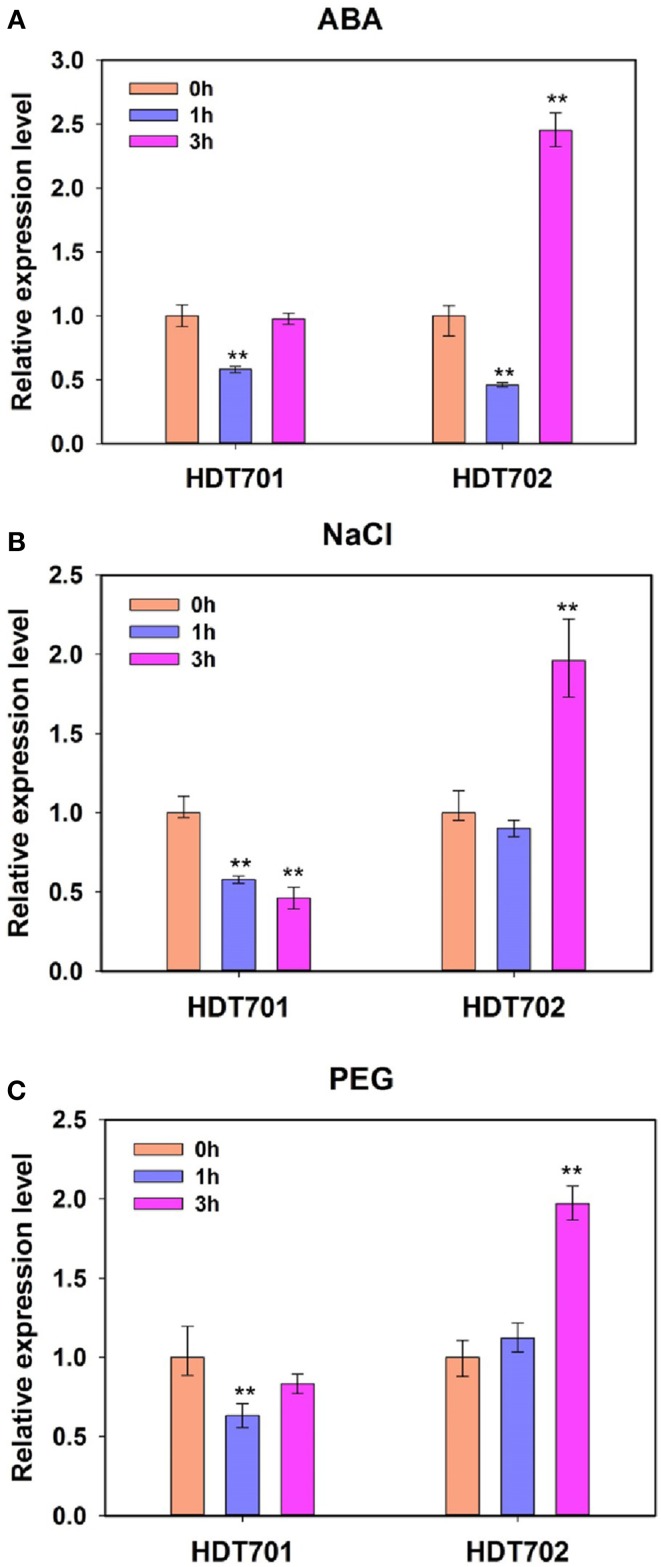
**Expression patterns of rice *HDT701* and *HDT702* under ABA, salt, and PEG stresses**. Two-week-old rice seedlings were treated with 100 μ M ABA **(A)**, 300 mM NaCl **(B)**, and 20% PEG **(C)** for 0, 1, and 3 h, respectively. *ACTIN* was used as the internal control. Eighteen seedlings were used for analysis for each treatment. Values are shown as means + SD (*t*-test: ^**^P < 0.01, difference from WT).

We further studied the expression patterns of *HDT701* and *HDT702* under ABA, salt and osmotic stresses. Here, 300 mM sodium chloride (NaCl) and 20% polyethylene glycol 6000 (PEG) were used to simulate salt and osmotic stress, respectively. The expression levels of *HDT701* decreased significantly 1 h after ABA, NaCl and PEG treatments, but 3 h after ABA and PEG exposure, they recovered (Figure 1). Furthermore, the expression of *HDT702* was down-regulated 1 h after ABA treatment, but 3 h after ABA, NaCl, and PEG exposure, the expression levels were significantly higher (Figure 1). Together, this data suggests that the expression patterns of *HDT701* and *HDT702* may be regulated by abiotic stresses.

### GUS staining analysis of HDT701 expression patterns in rice organs

**Figure 2:**
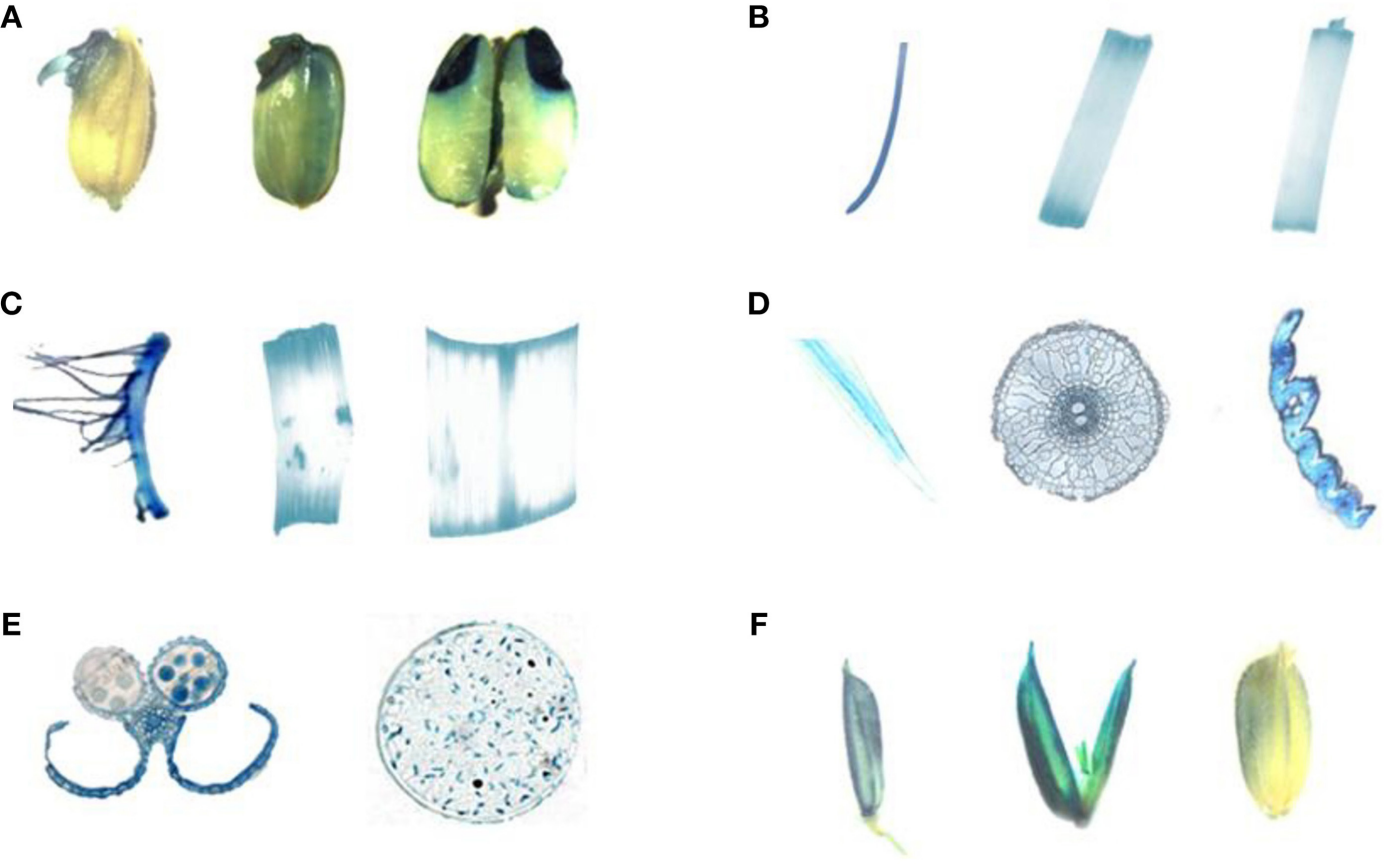
**GUS staining of HDT701 expression at various growth stages in rice**. **(A)** Germinating rice seeds. **(B)** Root, stem and leaf of 14-day-old seedling. **(C)** Root, stem, and leaf of 40-day-old rice plant. **(D)** Cross-section of root, stem, and leaf of 40-day-old rice plant. **(E)** Anther and pollen of 60-day-old rice plant. **(F)** Grains during maturation (85-day-old rice plant).

The expression patterns of HDT701 were then examined by using transgenic rice plants expressing β-glucuronidase (GUS) driven by the *HDT701* promoter. A strong GUS signal was detected in germinating rice seeds (Figure 2A), 14 and 40-day-old roots, petioles and leaves (Figures 2B–D). GUS staining was also detected in anthers, pollen (Figure 2E), and in the pollen grains during the maturation stage (Figure 2F). These data suggest that *HDT701* is constitutively expressed throughout the life cycle of rice.

### Subcellular localization of HDT701

**Figure 3:**
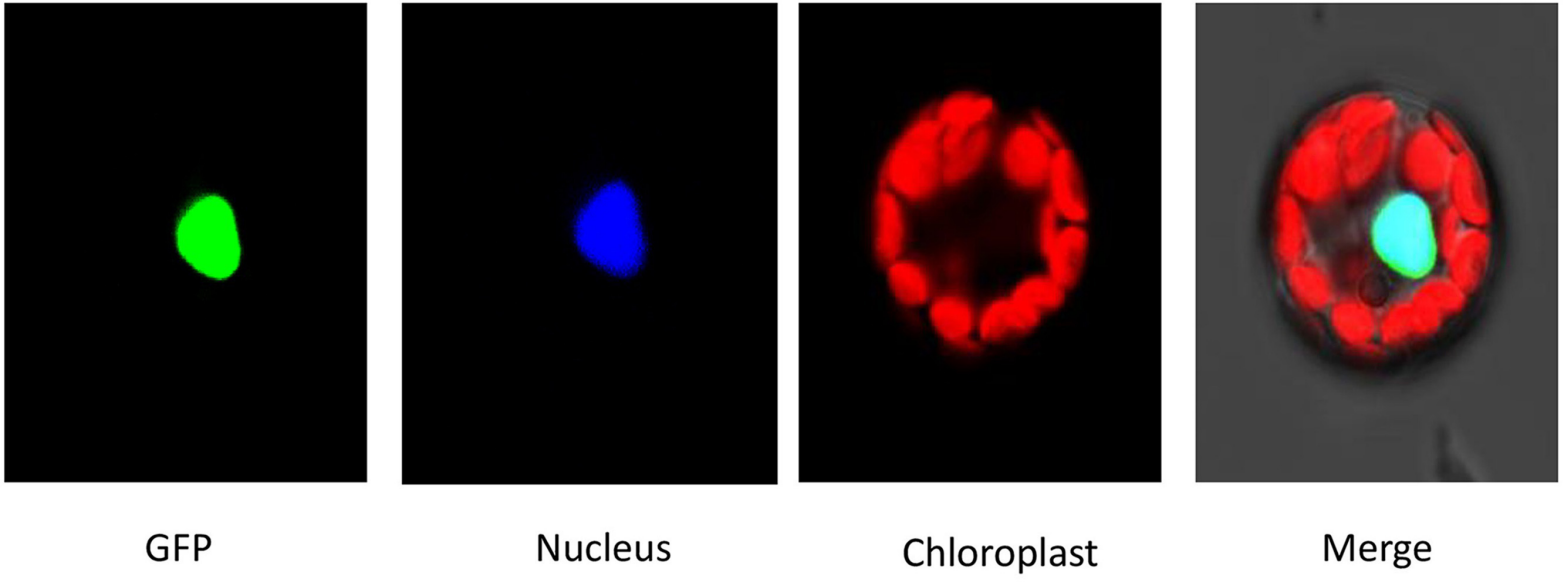
**Subcellular localization analysis of HDA701**. The constructs were transformed into *Arabidopsis* protoplast by PEG. mCherry was used as a nuclear marker.

To investigate the subcellular localization of HDT701 proteins, the coding region of *HDT701* fused with green fluorescent protein (GFP) was transformed into *Arabidopsis* protoplasts by PEG. Consistent with a previous report (Ding et al., 2012), we showed that HDT701-GFP is localized at a subcellular level in the nucleus of the protoplasts by a fluorescence microscopy assay (Figure 3).

### Generation and identification of rice *HDT701* overexpression and RNAi plants

**Figure 4:**
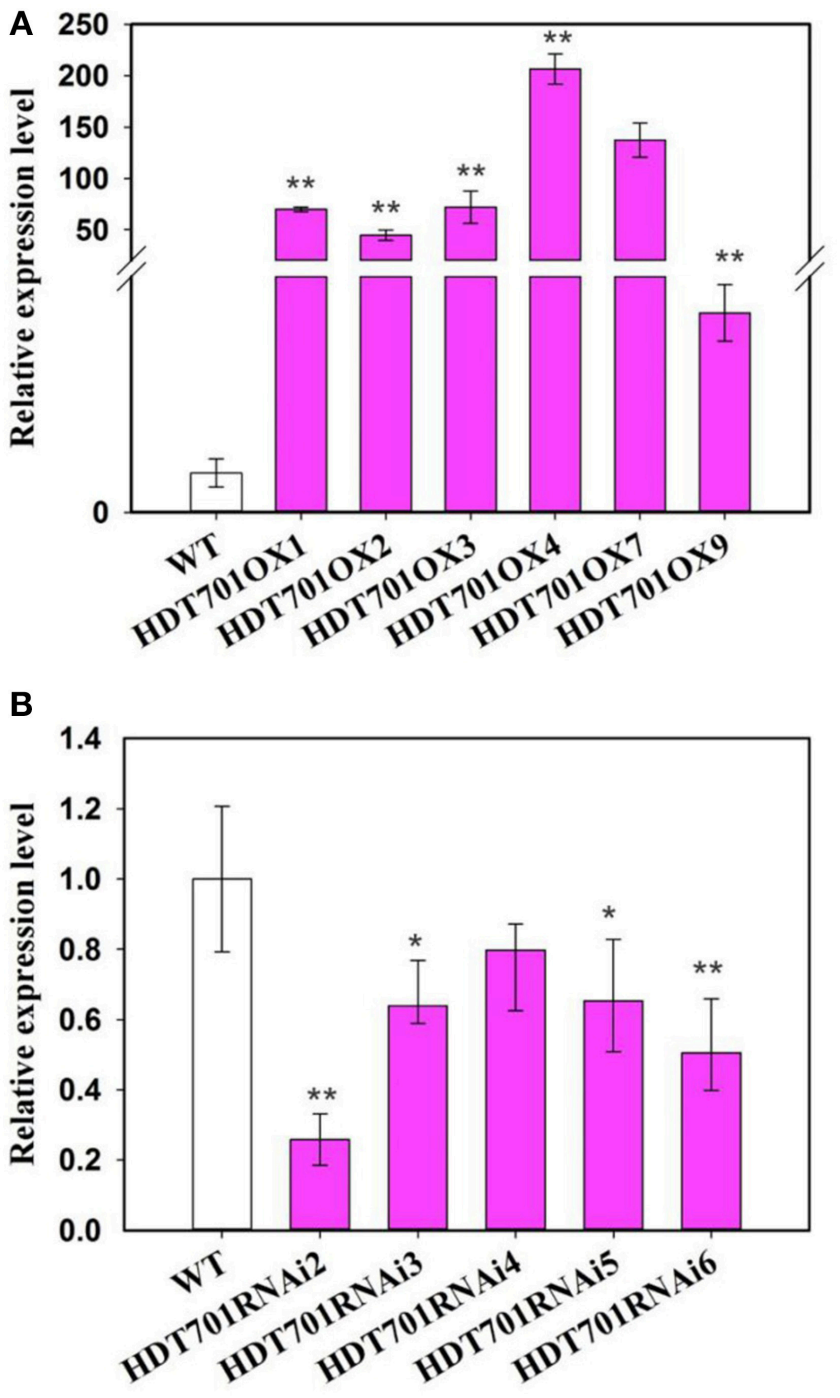
**Transcription an alysis of *HDT701OX* plants (A) and RNAi plants (B) by quantitative RT-PCR assay**. *ACTIN* was used as the internal control. At least 18 seedlings were used for analysis. Values shown as means + SD (*t*-test: ^*^P < 0.05, ^**^P < 0.01, difference from WT).

To elucidate the function of *HDT701* in rice, an OX construct was made in which the full length *HDT701* cDNA was expressed under the control of the maize *ubiquitin* promoter. The construct was transformed into rice via *Agrobacterium tumefaciens*-mediated transformation. Quantitative RT-PCR analysis confirmed increased expression of *HDT701* in five independent *HDT701OX* homozygous transgenic lines (Figure 4A). Two transgenic lines (*HDT701 OX4* and *OX7*) with high expression of *HDT701* were selected for further studies. Furthermore, we also generated *HDT701* RNAi transgenic plants using an RNAi construct, which was designed to target the specific 5' untranslated region (UTR) of *HDT701* under the control of the maize *ubiquitin* promoter. Quantitative RT-PCR analysis showed that the expression of *HDT701* was down-regulated in *HDT701 RNAi2* and *RNAi6* transgenic lines (Figure 4B).

### Overexpression of *HDT701* in rice delays seed germination under ABA, salt, and osmotic stresses

**Figure 5:**
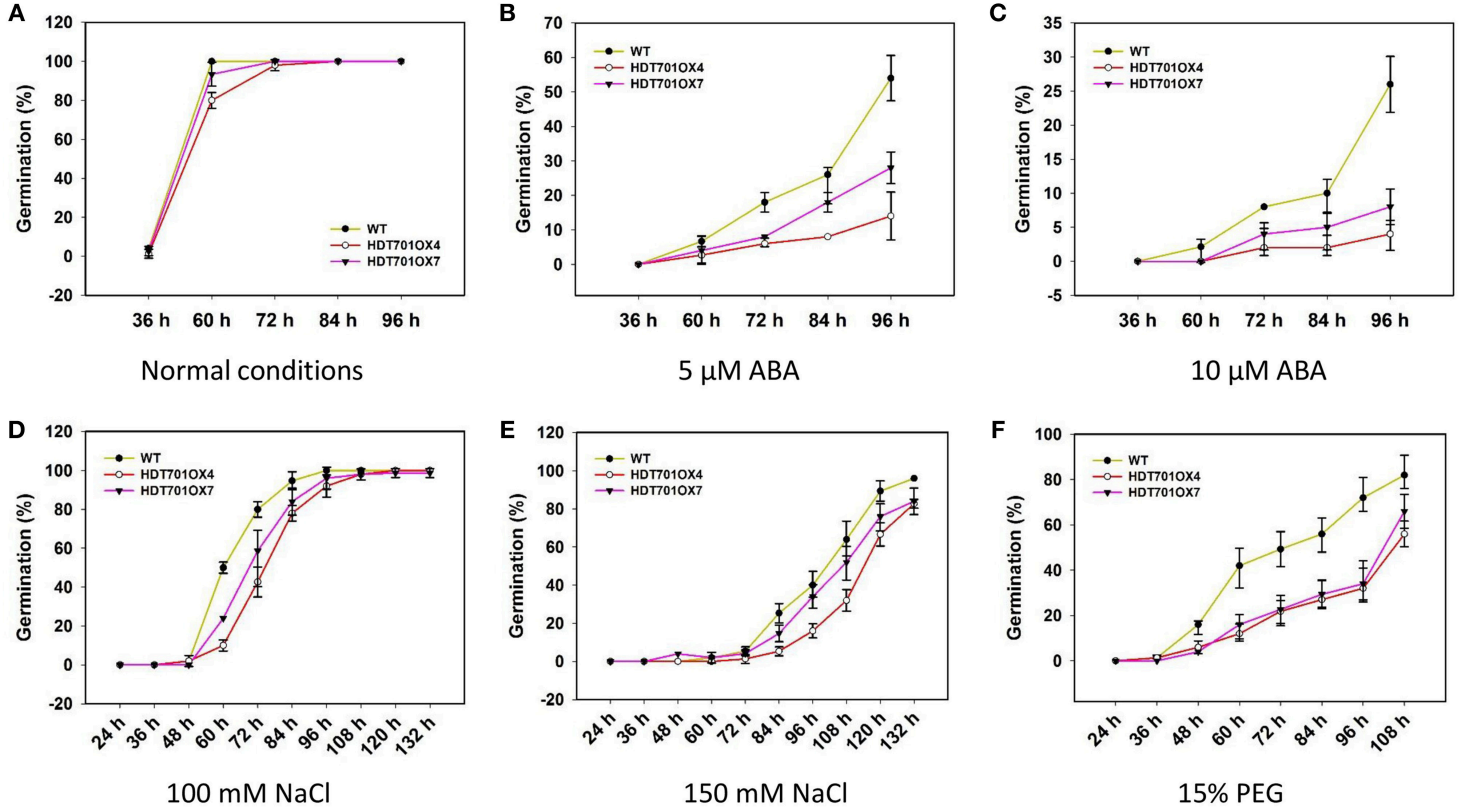
**Time course of seed germination of *HDT701 OX4* and *OX7* under normal conditions (A), 5 and 10 μM ABA (B,C), 100 and 150 mM NaCl (D,E), and 15% PEG (F)**. Hundred seeds were used for the analysis of each treatment.

Previous studies revealed that *Arabidopsis* HD2-type HDACs, namely HD2A, HD2B, HD2C and HD2D, are key regulators in stress responses (Liu et al., 2014), which prompted us to examine any possible role of rice HDT701 in responding to abiotic stresses. We compared the germination rates of *HDT701 OX* seeds with wild-type (WT) when separately treated with various concentrations of ABA, NaCl and PEG, as stressors. Relatively lower germination rates were detected for *HDT701 OX4* and *OX7* seeds 60 h after plating in medium compared to the WT under normal (control) conditions (Figure 5A), suggesting that overexpression of *HDT701* in rice may delay seed germination. Furthermore, the germination of *HDT701 OX4* and *OX7* seeds was significantly delayed in response to 5 and 10 μ M ABA compared to the WT (Figures 5B,C). Similarly, the germination of *HDT701* *OX4* and *OX7* seeds was also slower than the WT after treatment with 100 and 150 mM NaCl (Figures 5D,E). Upon exposure to 15% PEG, the germination rates of *HDT701 OX4* and *OX7* were obviously lower than the WT (Figure 5F). However, the germination rates of *HDT701 RNAi2* and *RNAi6* showed no obvious differences compared with the WT (Supplemental Figure 1). Collectively, these data indicate that overexpression of *HDT701* in rice may delay seed germination under ABA, salt and osmotic stresses.

### Overexpression of *HDT701* decreases gene expression and histone H4 acetylation levels of GA biosynthetic genes during seed germination

**Figure 6:**
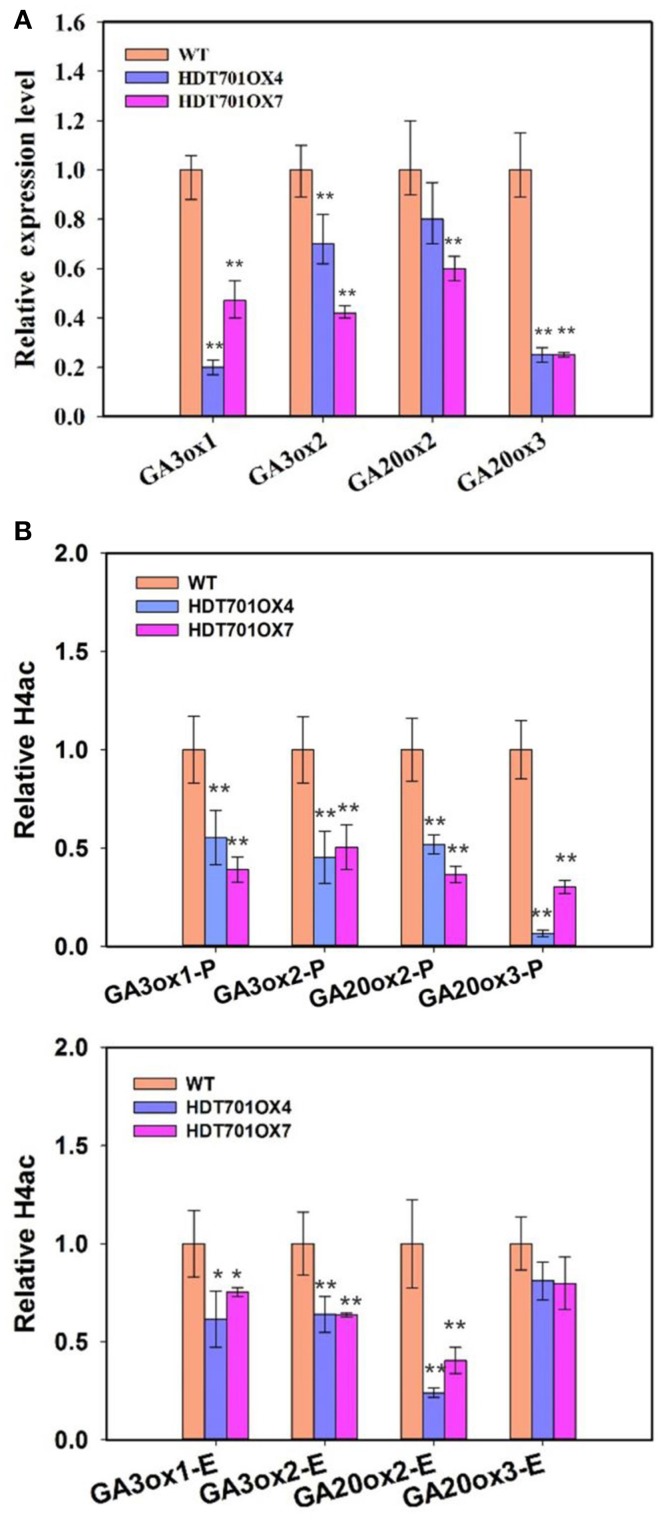
**Transcription and histone acetylation levels of GA biosynthesis genes in *HDT701* overexpression lines during seed germination**. **(A)** qRT-PCR analysis of the expression levels of GA biosynthetic genes, *GA3ox1*, *GA3ox2*, *GA20ox2*, and *GA20ox3* in *HDT701 OX4* and *OX7*. *ACTIN* was used as the internal control. **(B)** ChIP analysis of the histone H4 acetylation levels of the promoters and exons of GA biosynthetic genes in germinating *HDT701 OX4* and *OX7* seeds. P, promoter; E, exon. *UBQ* was used as the internal control. At least 30 seedlings of WT, *HDT701 OX4*, and *OX7* were used for analysis. Values are shown as means + SD (*t*-test: ^*^P < 0.05, ^**^P < 0.01, difference from WT).

Previous studies revealed that a plant hormone, gibberellin (GA), plays a key role in the control of seed germination (Ogawa et al., 2003). We further detected the transcription levels of the GA metabolic genes in germinating *HDT701* OX seeds. The expression levels of GA biosynthetic genes, *GA3ox1* and *GA3ox2*, *GA20ox2* and *GA20ox3*, were significantly down-regulated in *HDT701 OX4* and* OX7* seeds (Figure 6A).

Recent work has shown that HDT701 is a histone H4 deacetylase (Ding et al., 2012). We further examined the histone H4 acetylation levels of these GA biosynthetic genes in germinating *HDT701 OX* seeds by chromatin immunoprecipitation (ChIP) assays. There was an obvious decrease in histone H4 acetylation levels at promoter and exon regions of *GA3ox1*, *GA3ox2*, and *GA20ox2*, and the promoter region of *GA20ox3* in *HDT701 OX4* and *OX7* seeds compared with the WT (Figure 6B). This data suggests that HDT701 may repress the expression of GA biosynthetic genes by decreasing their histone H4 acetylation levels in germinating rice seeds.

### Overexpression of *HDT701* in rice enhances salt and osmotic resistance during the seedling stage

**Figure 7:**
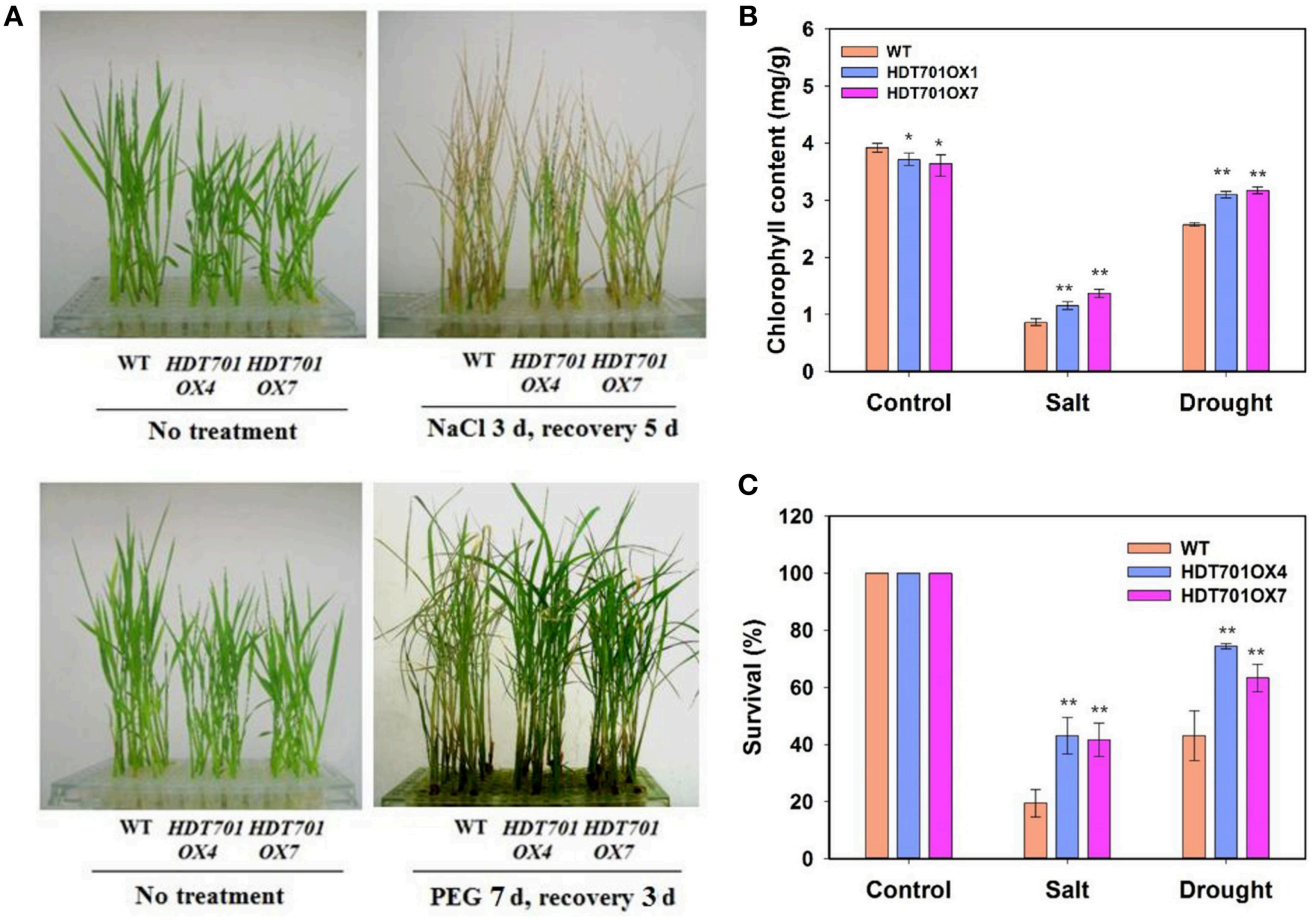
**Overexpression of *HDT701* enhances salt and osmotic stress tolerance**. **(A)** Phenotype of *HDT701 OX* seedlings after PEG and NaCl treatment. Two-week-old WT, *HDA701 OX4*, and *OX7* plants were treated with 150 mM NaCl or 20% PEG for indicated days and then recovered in MS medium. **(B)** Chlorophyll content of *HDT701 OX4* and *OX7* seedlings after NaCl and PEG treatments. **(C)** Survival rates of *HDT701 OX4* and *OX7* seedlings after NaCl and PEG exposure. Fifty seedlings were used for the analysis of each treatment. Values are shown as means + SD (*t*-test: ^*^P < 0.05, ^**^P < 0.01, difference from the WT).

We further examined the phenotype of *HDT701 OX* seedlings upon exposure to salt and osmotic stresses. Two-week-old *HDT701 OX* seedlings were treated with 150 mM NaCl or 20% PEG, respectively, and then recovered in MS medium for the indicated period. After recovery for 5 or 3 days, the NaCl- or PEG-treated *HDT701 OX* seedlings appeared less dehydrated than WT seedlings (Figure 7A). We also measured the chlorophyll content (Ming et al., 2007) of *HDT701 OX* seedlings, which was significantly higher than that of wild-type seedlings (Figure 7B). Furthermore, after recovery for 15 days, the survival rate of *HDT701 OX* seedlings was also higher than WT seedlings (Figure 7C). Taken together, this data indicates that overexpression of *HDT701* in rice may enhance salt and osmotic resistance during the seedling stage.

## Discussion

Previous studies have provided evidence for the involvement of *Arabidopsis* HDACs in ABA-related and other abiotic stresses (Yuan et al., 2013) In *Arabidopsis*, overexpression of *HD2C* plants demonstrated hyposensitivity to NaCl, in the form of inhibited seed germination and root growth (Sridha and Wu, 2006), while the loss of function of *HDA6* and *HDA19* mutants displayed a phenotype that is hypersensitive to NaCl and ABA stresses during seed germination in *Arabidopsis* (Chen and Wu, 2010; Chen et al., 2010). Down-regulation of *SRT1*, a SIR2 family *HDAC*, in rice enhanced tolerance to oxidative stress (Huang et al., 2007). In the present work, we showed that overexpression of *HDT701* in rice displayed increased sensitivity to ABA, NaCl, and PEG during germination, and enhanced tolerance to NaCl and PEG stresses of two-week-old rice seedlings. Taken together, these findings indicate that HDACs could be essential players in the response of rice plants to abiotic stress.

The N-terminal tails of the histone proteins can undergo a variety of posttranslational modifications, including acetylation, methylation, ubiquitination, phosphorylation, and sumoylation (Yuan et al., 2013). Recent work noted that the histone arginine demethylases, JMJ20 and JMJ22, act positively in regulating seed germination in *Arabidopsis*. JMJ20 and JMJ22 increased the expression of GA biosynthetic genes, *GA3ox1*, and *GA3ox2*, by removing the repressive histone arginine methylation of these genes through histone arginine demethylation activity (Cho et al., 2012). In the present study, overexpression of *HDT701* in rice decreased the expression levels of GA biosynthetic genes, *GA3ox1*, *GA3ox2*, *GA20ox2* and *GA20ox3*, and delayed seed germination, suggesting that HDT701 may negatively regulate seed germination by decreasing the levels of GA in rice seeds. In *Arabidopsis thaliana*, HDACs interact with various transcription factors to repress gene expression in multiple developmental processes (Liu et al., 2014). HDA15 specifically interacts with Phytochrome-Interacting Factor 3 (PIF3), a key transcription factor involved in photomorphogenesis, to repress chlorophyll biosynthetic gene expression in *Arabidopsis* (Liu et al., 2013). Furthermore, HDA6 associates with Asymmetric Leaves 1 (AS1) in repression of *KNOTTED-LIKE HOMOBOX* (*KNOX*) genes expression in control of leaf development in *Arabidopsis* (Luo et al., 2012b). HDT701 may also be associated with specific transcription factors to repress GA biosynthetic gene expression during rice seed germination. HDT701 was previously identified as a histone H4 deacetylase which directly bound to promoter regions of defense-related genes, *WRKY6* and *WRKY5* (Ding et al., 2012). The decrease in histone H4 acetylation levels of the GA biosynthetic genes in *HDT701 OX4* and *OX7* suggests that HDT701 may repress their expression through histone deacetylation. However, it is still unclear whether HDT701 is able to directly regulate the expression of these genes. Further genome-wide ChIP analysis may identify more direct target genes of HDT701, which may help to elucidate the role of HDT701 in plant development and in response to abiotic stresses.

## Materials and methods

### Plant materials

Rice (*Oryza sativa* L. var. Japonica) cv. Zhonghua 11 was used in this study. Plants were grown in a greenhouse in South China Botanical Garden, Guangzhou, China, at 25°C (16-h photoperiod, with a light extensity of 60–80 μmol m^−2^ s^−1^) from March to July. After harvest, on the 15th July, rice seeds were stored at −20°C (with 15% humidity) for 2 months before they were used for analysis.

### Bioinformatics analysis

The *cis*-acting elements in *HDT701* and *HDT702* promoters were analyzed using PlantCARE (http://bioinformatics.psb.ugent.be/webtools/plantcare/html/) (Lescot et al., 2002) and 2000-bp DNA sequences before the start codon were used for prediction.

### Plasmid constructs and rice transformation

To generate *GUS-HDT701* vectors, a 1884-bp fragment behind the transcriptional start site of *HDT701* was amplified and subcloned into the pCAMBIA 1381Z binary vector (Cambia. Canberra, Australia). To generate the *HDT701 OX* vector, the cDNA fragment of *HDT701* (accession number: AK072845) was amplified and then subcloned into binary vector pCU1301, which was modified based on pCAMIBA1301 (Cambia) and contained a maize (*Zea mays*) ubiquitin promoter. The primers used for vector construction are listed in Supplemental Table 2.

For transformation, at first, constructs were introduced into *Agrobacterium tumefaciens* strain EHA105 by freezing. Embryogenic callus from mature seeds of rice (“Zhonghua 11”) were used for transformation. The procedures for transformation, regeneration and transfer to soil were as described previously for rice (Hiei et al., 1994).

### Detection of *GUS* expression

Histochemical GUS assays were carried out according to the procedure of Jefferson et al. (1987). The transgenic plants were grown in a greenhouse at 25°C. Various organs including leaves, roots, seeds, germinating seeds, spikes, and flowers of transgenic rice were collected to examine GUS expression. These organs were immerged in GUS staining buffer (1 mM 5-bromo-4-chloro-3-indolyl-β-D-glucuronide citation(X-gluc), 100 mM sodium phosphate buffer (pH 7.0), 2 mM potassium ferricyanide, 2 mM potassium ferrocyanide, and 0.1% Triton X-100) at 37°C overnight. The stained materials were washed in ethyl alcohol.

### Salt and osmotic stress treatment of rice seedlings

For salt and osmotic stress treatment, two-week-old seedlings growing in MS (Murashige and Skoog, 2006) medium were transferred to MS medium supplemented with 150 mM NaCl for 3 days and then recovered in MS medium for 5 days, or transferred to MS medium to which 20% PEG was added for 7 days and then recovered in MS medium for 3 days.

### Measurement of seed germination rate

For the germination assay, seeds were pre-soaked in a thermostatic culture box (Henan Brother Device and Equipment Co., Ltd., Henan, China) at 37°C for 3 days to break any possible dormancy. Seeds were surface-sterilized by incubating in 75% (v/v) ethanol for 1 min, treated with 0.1% (v/v) HgCl_2_ for 15 min, then washed with sterile distilled water three times. Surface-sterilized seeds were sown on MS plates supplemented with 5 and 10 μ M ABA, 100 and 150 mM NaCl, and 15% PEG, respectively. For normal conditions, the seeds were sown on MS plates and used as the control. Plates were placed in a greenhouse at 25°C under long-day conditions (16-h photoperiod). Each treatment was repeated at least three times.

### Quantitative RT-PCR analysis

For gene expression analysis, after pre-soaking in sterile water for 3 d, the seeds were incubated on MS plates for 12 h then harvested for RNA extraction. Total RNA was extracted with TRIZOL reagent as described by the manufacturer (Invitrogen). After DNase treatment, the first strand of cDNA was synthesized by M-MLV reverse transcriptase (Promega) using 2 μg of total RNA as the template. Real-time PCR was performed on an optical 96-well plate with an ABI7500 real-time PCR system (Applied Biosystems). Each sample was quantified at least in triplicate. The rice *Actin* gene (accession no: NM_001057621) was used as the internal control. The gene-specific primers used for qRT-PCR are listed in Supplemental Table 2.

### Chromatin immunoprecipitation assays

The chromatin immunoprecipitation assays were performed as described by Gendrel et al. (2005) with minor changes. Briefly, seeds were fixed with 1% formaldehyde in a vacuum for 1 h. Chromatin was extracted and sheared to an average length of 500 bp fragments by 6 repeats of 10 s sonication at 20% duty cycle and 1.5 power output using a Branson Sonifier 250, and then immunoprecipitated with an anti-acetyl-histone H4 antibody (Millipore; 06-866). Cross-linking was then reversed, and the amount of each precipitated DNA fragment was determined by quantitative PCR. *UBQ* (accession no: AC103891) was used as the internal control. Primers used for ChIP assays are listed in Supplemental Table 2.

### Conflict of interest statement

The Associate Editor, Yuhai Cui, and the Review Editors, Steve Robinson and Ming-Jun Gao, declare that, despite being affiliated to the same institution as the author Lining Tian, the review process was handled objectively and no conflict of interest exists. The authors declare that the research was conducted in the absence of any commercial or financial relationships that could be construed as a potential conflict of interest.

## Supplementary material

Supplemental Figure 1 **Seed germination rates of *HDT701 RNAi2* and *RNAi6* lines under normal conditions (A) 5 μM ABA (B) 100 mM NaCl (C), and 10% PEG (D)**.
