# Supplementary material for: Expression and functional analysis of the plant-specific histone deacetylase HDT701 in rice
Source: Front Plant Sci. 2015 Jan 20;5:764. doi: 10.3389/fpls.2014.00764 (PMC4299430; doi:10.3389/fpls.2014.00764)
Supplement: Supplementary file 1 [file Table1.DOC]

*Supplemental Table S1. Quantitative statistical analysis of cis-acting elements enriched in the 2 kb-upstream promoter regions of HDT701 and HDT702.*

| ***Cis*-element** | **Function** | **HDT701** | **HDT702** |
| --- | --- | --- | --- |
| **Stress-responsive elements** MYCCONSENSUSAT  ACGTATERD1  CURECORECR  WRKY71OS  GATABOX  INRNTPSADB  BIHD1OS  SORLIP1AT  **Tissue-specific elements**  DOFCOREZM  ROOTMOTIFTAPOX1  GTGANTG10  POLLEN1LELAT52  RYREPEATBNNAPA  **Hormone-responsive elements**  ABRE  ARR1AT  MYBCORE  WBOXATNPR1  TCA-element  CGTCA-motif  TGACG-motif | dehydration-responsive  early responsive to dehydration  copper-response element  pathogenesis-related  light-regulated  light-responsive  disease resistance responses  light-induced  endosperm-specific  activate root-specific expression  anther/pollen-specific expression  pollen- specific expression  developing plant embryos  ABA-responsive  cytokinin-regulated  ABA-independent  SA-induced  SA-responsive  JA-responsive  JA-responsive | 18  8  14  8  18  7  4  1  30  7  17  8  1  2  20  4  2  2  1  0 | 14  22  2  11  5  2  4  12  4  4  7  6  1  8  9  6  2  0  0  3 |
