# Supplementary material for: Expression and functional analysis of the plant-specific histone deacetylase HDT701 in rice
Source: Front Plant Sci. 2015 Jan 20;5:764. doi: 10.3389/fpls.2014.00764 (PMC4299430; doi:10.3389/fpls.2014.00764)
Supplement: Supplementary file 2 [file Table2.DOC]

| **Primers used for construction of *HDT701* GUS-staining vectors** | |
| --- | --- |
| HDT701 GUS pr1 | 5′-TGAAGCTTTAAGGCGAATAAGCGAAAC-3′ |
| HDT701 GUS pr2 | 5′-CTCTGCAGGAAGAACCCTAGAAAAGAAA-3′ |
|  |  |
| **Primers used for construction of *HDA701 OX* and *HDA701 RNAi* plants** | |
| HDT701 OX pr1 | 5′-CGGGATCCATGGAGTTCTGGGGTCTTGA-3′ |
| HDT701 OX pr2 | 5′-CGGGATCCTCACTTGGCGGGGTGCTTGG-3′ |
| HDA701 RNAi pr1 | 5′-CGGGATCGAGCTCAAGGAAAATGAGCAGAAAAA-3′ |
| HDA701 RNAi pr2 | 5′-GGGGTACCACTAGTTTGGCGGGGTGCTTGGCCTT-3′ |
|  |  |
| **Primers used for qRT-PCR assay** | |
| Actin-F | 5′-GATTGCCAAGGCTGAGTACGA-3′ |
| Actin-R | 5′-AAAGAGAGAAACAAGCAGGAGGA-3′ |
| GA3ox1-F | 5′-CGACGAGTTGCTGAGGTT-3′ |
| GA3ox1-R | 5′-AGGTGAAGAAGCCCGAGT-3′ |
| GA3ox2-F | 5′-ATGCCCTACTTCCTCGGT-3′ |
| GA3ox2-R | 5′-CTTGTCCTCTTCCTTCGCTA-3′ |
| GA20ox2-F | 5′-TCGCTGACGATCATGGAACT-3′ |
| GA20ox2-R | 5′-CATGATTGAGCTGCTGTCCG-3′ |
| GA20ox3-F | 5′-AGAGGCACTACCGGTCGGA-3′ |
| GA20ox3-R | 5′-TCGAGGCTTTCATAGCCATTCC-3′ |
|  |  |
| **Primers used for ChIP-qPCR assay** | |
| CHIP-UBQ-F | 5′-TTCCACATCCTTCTCTACGGC-3′ |
| CHIP-UBQ-R | 5′-ATGGGTGTTTTCACTGACATGG-3′ |
| CHIP-GA3ox1-PF | 5′-CCCTCTGTCCCAATCACCAC-3′ |
| CHIP-GA3ox1-PR | 5′-AGGGAGAGAAGTTTTGGAGCG-3′ |
| CHIP-GA3ox1-EF | 5′-ACATATATACTCTCCCATGCATTCC-3′ |
| CHIP-GA3ox1-ER | 5′-AAACTCCTCCATCACGTCACT-3′ |
| CHIP-GA3ox2-PF | 5′-ATTTTGTGCCGAACGATGCG-3′ |
| CHIP-GA3ox2-PR | 5′-AACGCACAAAAGCCTGTGAC-3′ |
| CHIP-GA3ox2-EF | 5′-GATCGATCCGCCATTGCTTG-3′ |
| CHIP-GA3ox2-ER | 5′-CCGAGCGCCTTGAAGAACAT-3′ |
| CHIP-GA20ox2-PF | 5′-GGGGGTCATTGATTCGACCA-3′ |
| CHIP-GA20ox2-PR | 5′-GCAGGAGGGTGGGGTATTTG-3′ |
| CHIP-GA20ox2-EF | 5′-GATGAAGGAGCTGTCGCTGA-3′ |
| CHIP-GA20ox2-ER | 5′-CGCATGATTGAGCTGCTGTC-3′ |
| CHIP-GA20ox3-PF | 5′-TCATTGTGGATCCGGGTGAC-3′ |
| CHIP-GA20ox3-PR | 5′-GTGGTGGTTTGTTATGGCCG-3′ |
| CHIP-GA20ox3-EF | 5′-ACACCTGGCTCCGAGTTCTA-3′ |
| CHIP-GA20ox3-ER | 5′-TAAGAAGGCCTCGTGTGGAA-3′ |

**Supplemental Table S2.** Primer pairs used in this study
