# Supplementary figures and images for: Expression and functional analysis of the plant-specific histone deacetylase HDT701 in rice
Source: Front Plant Sci. 2015 Jan 20;5:764. doi: 10.3389/fpls.2014.00764 (PMC4299430; doi:10.3389/fpls.2014.00764)

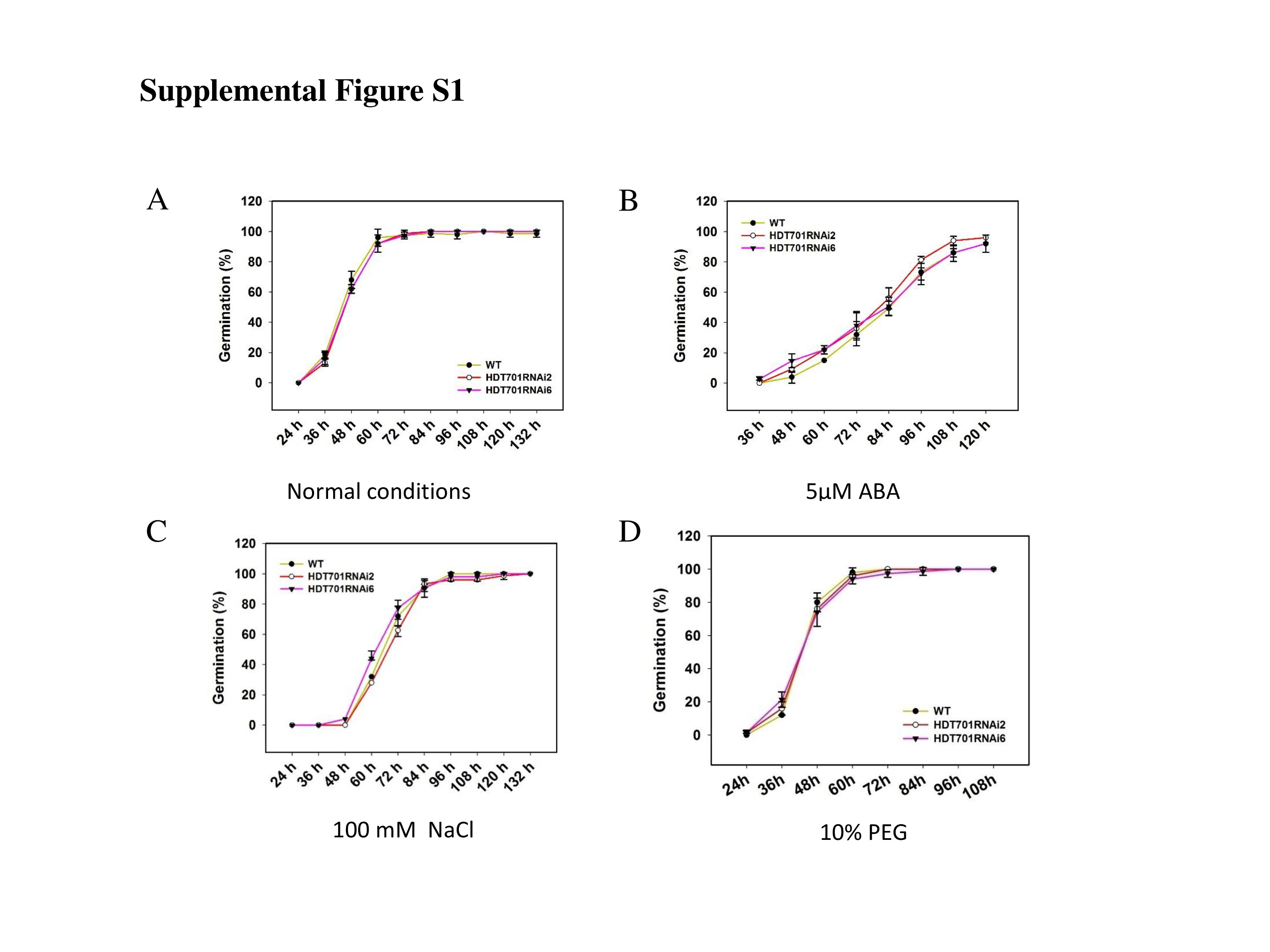

Supplement: Supplemental Figure 1 — Seed germination rates of HDT701 RNAi2 and RNAi6 lines under normal conditions (A) 5 μM ABA (B) 100 mM NaCl (C), and 10% PEG (D). [file Image1.JPEG]
